# Anti-Anxiety Effect of (−)-Syringaresnol-4-*O*-β-d-apiofuranosyl-(1→2)-β-d-glucopyranoside from *Albizzia julibrissin* Durazz (Leguminosae)

**DOI:** 10.3390/molecules22081331

**Published:** 2017-08-11

**Authors:** Jie Liu, Yue-Wei Lv, Jin-Li Shi, Xiao-Jie Ma, Yi Chen, Zhi-Quan Zheng, Sheng-Nan Wang, Jian-You Guo

**Affiliations:** 1 School of Chinese Materia Medica, Beijing University of Chinese Medicine, 11ANorth Third Ring East Road, Chaoyang District, Beijing 100029, China; liujiealice@163.com (J.L.); lvyuewei100@126.com (Y.-W.L.); 20150931927@bucm.edu.cn (X.-J.M.); chenyi861101@sina.com (Y.C.); zqzheng@bucm.edu.cn (Z.-Q.Z.); Wasnan@sina.com (S.-N.W.); 2 Key Laboratory of Mental Health, Institute of Psychology, Chinese Academy of Sciences, 4ADatun Road, Chaoyang District, Beijing 100101, China

**Keywords:** (−)-syringaresnol-4-*O*-β-d-apiofuranosyl-(1→2)-β-d-glucopyranoside, open field test, elevated maze plus, HPA axis, monoaminergic systems

## Abstract

*Albizzia julibrissin* Durazz, a Chinese Medicine, is commonly used for its anti-anxiety effects. (−)-syringaresnol-4-*O*-β-d-apiofuranosyl-(1→2)-β-d-glucopyranoside (SAG) is the main ingredient of *Albizzia julibrissin* Durazz. The present study investigated the anxiolytic effect and potential mechanisms on the HPA axis and monoaminergic systems of SAG on acute restraint-stressed rats. The anxiolytic effect of SAG was examined through an open field test and an elevated plus maze test. The concentration of CRF, ACTH, and CORT in plasma was examined by an enzyme-linked immune sorbent assay (ELISA) kit while neurotransmitters in the cerebral cortex and hippocampus of the brain were examined by High Performance Liquid Chromatography (HPLC). We show that repeated treatment with SAG (3.6 mg/kg, p.o.) significantly increased the number and time spent on the central entries in the open-field test when compared to the vehicle/stressed group. In the elevated plus maze test, 3.6 mg/kg SAG could increase the percentage of entries into and time spent on the open arms of the elevated plus maze. In addition, the concentration of CRF, ACTH, and CORT in plasma and neurotransmitters (NE, 5-HT, DA and their metabolites 5-HIAA, DOPAC, and HVA) in the cerebral cortex and hippocampus of the brain were decreased after SAG treatment, as compared to the repeated acute restraint-stressed rats. These results suggest that SAG is a potential anti-anxiety drug candidate.

## 1. Introduction

Neuropsychiatric disorders are expected to rise sharply [1]. Anxiety disorders are the most common mental disorders, affecting nearly one in five adults in the U.S. alone [2]. The development of anxiolytic drugs is important for the treatment of anxiety disorders. Benzodiazepines have been used for the treatment of several forms of anxiety, but these compounds have well-known side effects, including sedation, muscle relaxation, amnesia, and dependence potential [3]. *Albizzia julibrissin* Durazz (Leguminosae, the cortex of *Albizia julibrissin* Durazz. officially recognized in the Chinese Pharmacopoeia), commonly named mimosa or silk trees, are widely distributed in Asia. Asians administered *A. julibrissin* soup to patients as a folk medicine to treat insomnia, diuresis, sthenia, and confusion [4]. The extract of *A. julibrissin* has an anxiolytic-like effect due to 5-HT_1A_ receptor activation, and because it has no unwanted adverse effects [5]. (−)-Syringaresnol-4-*O*-β-d-apiofuranosyl-(1→2)-β-d-glucopyranoside (SAG) is the main ingredient of the cortex of *A. julibrissin*, and we speculate that it may be the effective component in treating anxiety disorders [6]. 

Monoamine neurotransmitters including 5-HT, norepinephrine (NE), and dopamine (DA) are believed to be involved in pathogenes is of emotional disorders, and play an important role in mediating behavioral effects [7]. Several preclinical and clinical reports provided evidence to support that a dysfunction of the monoaminergic system may be implicated as a promising mechanism in the pathophysiology of anxiety disorders [8,9,10].Furthermore, anxiety disorders were closely related to the hippocampus and cerebral cortex of the brain. Meanwhile, a growing body of evidence suggests connections between dysregulation of the hypothalamic-pituitary-adrenocortical (HPA) axis and affective behaviors [11]. For example, hyperactivity of the HPA axis [12], enlarged adrenal glands, altered daily rhythm of corticoids secretion, and impairments in the HPA negative feedback mechanism have been implicated in emotional disorders. It is well known that HPA axis activation is a key component of the physiological response to stress and anxiety [13], while the monoamine neurotransmitters in the cerebral cortex, such as NE, DA and 5-HT, and plasma CORT are considered to be involved in generating symptoms of anxiety [14]. 

Stress, including chronic and acute restraint stress, is the most important and widely used animal model of anxiety [15]. Stress can induce behavioral, physiological, cognitive, and neural changes, potentially altering homeostasis and promoting vulnerability to illness [16]. Acute stress is an adaptive response to a multitude of adverse stimuli that are perceived to be noxious or threatening [17]. Acute restraint stress (ARS) can induce both psychological and physical effects. This results in a broad range of behavioral and physiological changes, including anxiogenic-like effects [18], endocrine, and autonomic alterations [19]. Continued stressful situations can be responsible for activation of the hypothalamic-pituitary-adrenal (HPA) axis, which subsequently results in the development of anxiety and depression [20]. 

In the present study, we examined the anxiolytic-like effect of SAG on repeated acute restraint-stressed rats using the elevated plus maze and open field test. Furthermore, in order to explore the potential mechanisms on monoaminergic systems and the HPA axis, we also examined the influences on the levels of the plasma corticotropin releasing factor (CRF), adrenocortico tropic hormone (ACTH), and corticosterone (CORT). We also studied the level of monoamines noradrenaline (NE), dopamine (DA), serotonin (5-HT) and their metabolites 5-HIAA, DOPAC, and HVA in the whole of the cerebral cortex and hippocampus of rats.

## 2. Results

### 2.1. Effect of SAG in the Open Field Test

**Figure 1:**
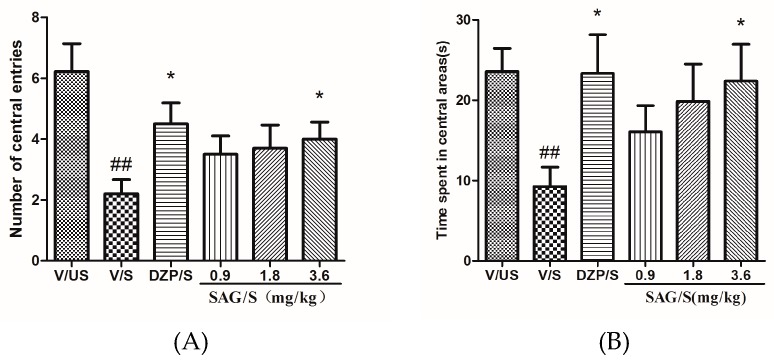
Effect of (−)-syringaresnol-4-*O*-β-d-apiofuranosyl-(1→2)-β-d-glucopyranoside (SAG) on the number of central entries and the time spent in central areas in the open field test in rats. (**A**) Number of central entries; (**B**) Time spent in central areas. Bars represent mean ± SEM. ## *p* < 0.01 vs. vehicle/unstressed group; * *p* < 0.05 vs. vehicle/stressed group. One-way ANOVA with Student-Newman-Keuls post hoc test.

The results of the OFT are shown in Figure 1 (Supplementary Materials). There were significant differences in the number of central entries (F(5,53) = 3.743, *p* < 0.01), and time spent in central areas (F(5,53) = 2.637, *p* < 0.05). Following the ARS, the number of central entries (*p* < 0.01) and the time spent in central areas (*p* < 0.01) of rats in the V/S group significantly decreased when compared with the V/US group. Compared with the V/S group, diazepam, and SAG (3.6 mg/kg) could significantly increase the number of central entries (*p* < 0.05) and the time spent in central areas (*p* < 0.05). However, there was no difference with SAG at the doses of 0.9 mg/kg and 1.8 mg/kg.

### 2.2. Effect of SAG in the Elevated Plus Maze

**Figure 2:**
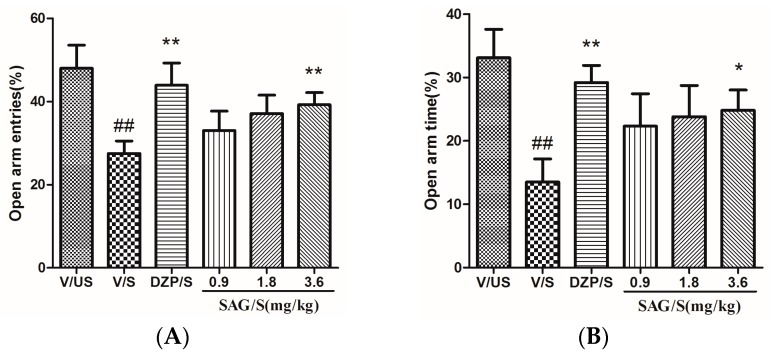
Effect of SAG on the open arm entries and the percentage of time spent in open arms in the elevated plus maze in rats. (**A**) Open arm entries; (**B**) Percentage of time spent in open arms. Bars represent mean ± SEM. ## *p* < 0.01 vs. vehicle/unstressed group; * *p* < 0.05 or ** *p* < 0.01 vs. vehicle/stressed group. One-way ANOVA with Student-Newman-Keuls post hoc test.

As shown in Figure 2, there were significant differences among groups in the percentage of open arm entries (F(5,53) = 2.751, *p* < 0.05) and the time spent on the open arms (F(5,53) = 2.602, *p* < 0.05). The V/S group significantly decreased the percentage of open arm entries (*p* < 0.01) and the time spent on the open arms (*p* < 0.01) of rats compared with the V/US group. Compared to the V/S group, diazepam and SAG (3.6 mg/kg) could significantly increase the percentage of open arm entries (both *p* < 0.01) and the time spent on the open arms (*p* < 0.01, *p* < 0.05) (Supplementary materials are available online).

### 2.3. Effect of SAG on Serum CRF, ACTH, and CORT Levels

**Figure 3:**
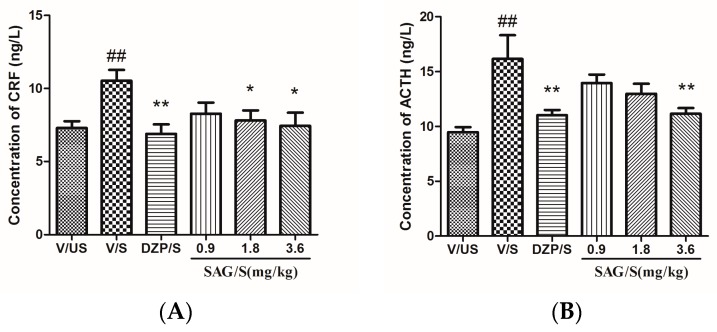

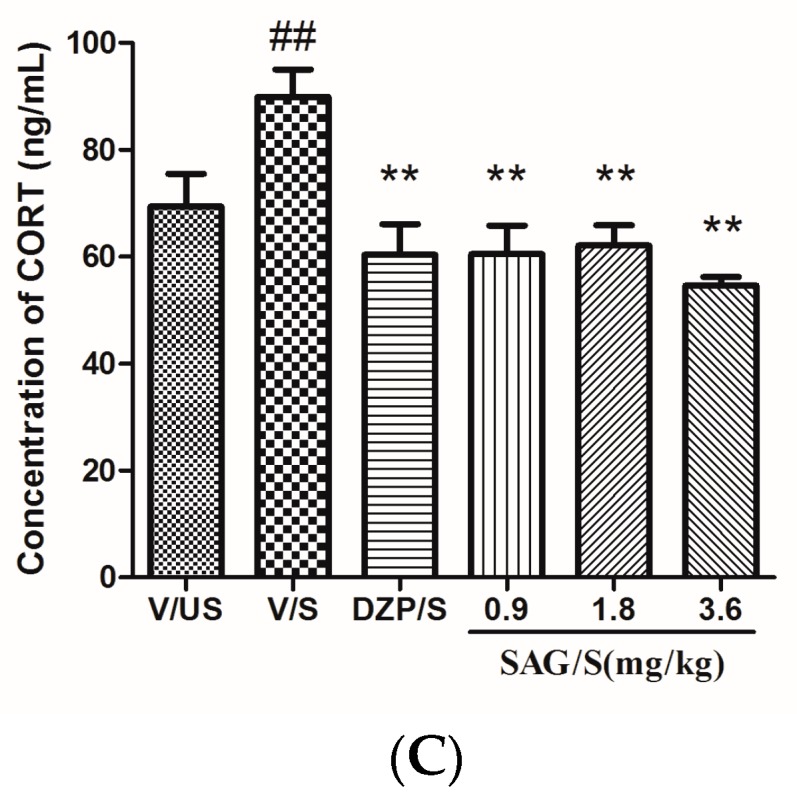
Effect of SAG (0.9, 1.8, 3.6 mg/kg) on serum CRF, ACTH and CORT levels after the behavior tests. (**A**) Concentration of CRF (ng/L); (**B**) Concentration of ACTH (ng/L); (**C**) Concentration of CORT (ng/mL). Bars represent mean ± SEM. ## *p* < 0.01 vs. vehicle/unstressed group; * *p* < 0.05 or ** *p* < 0.01 vs. vehicle/stressed group. One-way ANOVA with Student-Newman-Keuls post hoc test.

As shown in Figure 3, the one-way ANOVA revealed significant differences in the concentration of CRF (F(5,54) = 3.377, *p* < 0.05), ACTH (F(5,54) = 5.092, *p* < 0.01), and CORT (F(5,54) = 6.625, *p* < 0.01). With the acute restraint stress, the V/S group significantly increased the content of plasma CRF, ACTH, and CORT compared with the V/US group (all *p* < 0.01). Diazepam treated rats could significantly decrease the plasma CRF, ACTH, and CORT level compared with the V/S group (all *p* < 0.01). SAG at the doses of 1.8 and 3.6 mg/kg could significantly decrease the plasma CRF level (*p* < 0.05). The level of ACTH could be significantly decreased by SAG at the dose of 3.6 mg/kg, and the level of CORT could be significantly decreased by SAG at the doses of 0.9, 1.8, and 3.6 mg/kg. (Supplementary materials are available online).

### 2.4. Effect of SAG on Monoamine Neurotransmitters and Their Metabolites

In Table 1, the one-way ANOVA indicated significant differences in the levels of monoamine neurotransmitters and their metabolites in the cerebral cortex of rats (F(5, 53) = 12.561, *p* < 0.01, NE; F(5,53) = 3.065, *p* < 0.05, DA; F(5, 53) = 2.562, *p* < 0.05, HVA; F(5, 53) = 8.324, *p* < 0.01, 5-HT; F(5,53) = 11.435, *p* < 0.01, 5-HIAA. No differences were found in the level of DOPAC (F(5,53) = 1.258, *p* > 0.05). The V/S group could significantly increase the levels of NE (*p* < 0.01), DA (*p* < 0.05), HVA (*p* < 0.05), 5-HT (*p* < 0.05) and 5-HIAA(*p* < 0.05) when compared with the V/US group. SAG at the doses of 1.8 and 3.6 mg/kg could significantly decrease the levels of NE (*p* < 0.05, *p* < 0.01) and 5-HT (*p* < 0.05, *p* < 0.01). At the dose of 3.6 mg/kg, SAG could significantly decrease the levels of DA (*p* < 0.05), HVA (*p* < 0.05) and 5-HIAA (*p* < 0.01). (Supplementary materials are available online).

In Table 2, the one-way ANOVA indicated significant differences in the levels of monoamine neurotransmitters and their metabolites in the hippocampus of rats (F(5,53) = 3.716, *p* < 0.01, NE; F(5,53) = 4.879, *p* < 0.05, 5-HT; F(5,53) = 2.656, *p* < 0.05, 5-HIAA). No differences were found in the levels of DA (F(5,53) = 1.421, *p* > 0.05), DOPAC (F(5,53) = 1.624, *p* > 0.05) and HVA (F(5,53) = 1.245, *p* > 0.05). The V/S group could significantly increase the levels of NE (*p* < 0.01), 5-HT (*p* < 0.05) and 5-HIAA (*p* < 0.05) compared with the V/US group. SAG at the doses of 1.8 and 3.6 mg/kg could significantly decrease the levels of NE (both *p* < 0.01) and 5-HT (both *p* < 0.01). At the dose of 3.6 mg/kg, SAG could significantly decrease the level of 5-HIAA (*p* < 0.01). (Supplementary materials are available online).

## 3. Discussion

The results of this behavioral investigation revealed the anxiolytic-like effect of SAG in animal models of anxiety. Moreover, the results support the designed hypothesis that the HPA axis and monoaminergic systems play an important role in pathogenesis of anxiety disorders. Repeated ARS-subjected rats exhibited anxiety-like behavior. This is evidenced by the significant decline of central entries and time spent in central areas of OFT, the open arm entries, and time spent in open arm in EPM. Treatment with SAG at the dose of 3.6 mg/kg prevented the decrease resulting from the ARS by regulating the levels of CRF, ACTH, and CORT in serum and monoamine neurotransmitters and their metabolites.

Stress refers to any given condition affecting the integrity of biological systems. Since acute stress is more related with the expression of adaptive responses, it is characterized by early compensatory responses oriented to restore homeostatic conditions, and it provides relevant information on the origin and nature of ongoing harmful events in the nervous system. Stress is often associated with psychiatric (depression, anxiety, and panic) and neurodegenerative disorders (Alzheimer’s disease, Parkinson’s disease, etc.). Earlier reports have suggested that animals submitted to acute RS for 3 h induced long lasting anxiety-like behavior in the EPM [21] and acute RS for 60 min could activate the AT1-angiotensin receptors in the PVN [22], which is related to anxiety-like behavior in the brain. Of particular interest to this study is the aim to investigate the anti-anxiety effect of SAG in repeated acute restraint-stressed rats. In the present study, the procedure of repeated ARS is improved on Ortiz (1996) [23], and is verified by Lv (2015) [24]. Our findings show that repeated acute restraint stress could lead to anxiety and interfere with the HPA axis.

The dried stem bark of *A. julibrissin* has been used in China, Japan and Korea as a tonic, to ease the state of mind and calm the nerves [25]. Previous work has reported that the water extract [5] and n-butyl alcohol extract of *A. julibrissin* have obvious anti-anxiety effects [6]. Flavonol glycoside isolated from *A. julibrissin* showed sedative activity in mice [26]. Kinjo et al. reported that syringesinol glycoside isolated from the *A. julibrissin* cortex had a tonic effect. It is inferred that the lignan might be the valid anti-anxiety effective components [6]. Meanwhile, the SAG is the main lignan of *A. julibrissin*. The present study has investigated the anxiolytic effect of SAG and determined which neuronal mechanism is primarily involved. Our results defined the anti-anxiety effect and interpret the mechanism of SAG.

The open field test is widely used to examine the behavioral effects of drugs and anxiety [27]. The test is based on rodents’ natural tendency to remain near the periphery of a novel environment, and their aversion to open and illuminated spaces. Activity in the central area of the open field is thought to be correlated with the degree of fear, whereas activity in the peripheral zone and along the walls of the apparatus is thought to reflect general activity [28]. To demonstrate anxiogenic or anxiolytic effects of specific treatments, the control animals should display a reasonably high number of entries into the aversive parts of the apparatus, and be active in the peripheral areas of the open field. Anxiety-like behavior is reflected by diminished exploration of the aversive space with relatively unaffected ambulatory activity in the safe areas [29]. Rats that were treated with 3.6 mg/kg SAG could significantly increase the number of central entries and the time spent in central areas. Therefore, 3.6 mg/kg of SAG appeared to exert significant anxiolytic-like effects in this paradigm.

The elevated plus maze is considered an ethologically valid animal model of anxiety because it uses natural stimuli (e.g., fear of novel open spaces and fear of balancing on a relatively narrow, raised platform) that can induce anxiety in humans [30]. The elevated plus maze is based on the natural aversion of rodents to height and open spaces. In the present study, oral administration of SAG could induce anxiolytic-like effects on repeated acute restraint-stressed rats by increasing the number of entries into, and time spent on the open arms of the elevated plus maze.

A prominent mechanism by which the brain reacts to acute and chronic stress is activation of the hypothalamic-pituitary-adrenal (HPA) axis. The activity of the HPA axis is controlled by several brain pathways, including the hippocampus (which exerts an inhibitory influence on hypothalamic CRF-containing neurons via a polysynaptic circuit) and the amygdala (which exerts a direct excitatory influence) [31]. CRF is a critical mediator of fear conditioning and other forms of emotional memory to both aversive and rewarding stimuli. The well documented hyperactivity of the HPA axis in depression seems to be related to hypothalamic secretion of CRF [32]. Acute and chronic stress can increase CRF levels in the locus coeruleus and anxiolytic drugs can reduce them [33]. The current study has shown that the repeated ARS rats have a high level of CRF, and SAG can reduce them. Furthermore, they also show an increase in basal ACTH and CORT in the repeated ARS rats. By comparison, varying alterations in ACTH and/or CORT have been reported in studies employing the widely-used Chronic Variable Stress (CVS)/Chronic Mild Stress (CMS) model. Most have shown either no change or an increase in basal ACTH [34] and no change or an increase in basal CORT [35]. Changes in HPA responses to acute stress challenge after CVS/CMS are variable. One study reported an increased ACTH response to acute restraint stress, but no change in CORT response. From the above, the present study revealed that ARS could significantly increase the CRF, ACTH and CORT levels, and this effect could be retrained by DZP and SAG (3.6 mg/kg).Furthermore, SAG at the dose of 1.8 mg/kg could also reverse the increase of CRF and CORT caused by ARS. SAG at the dose of 0.9 mg/kg only could decrease the level of CORT. Our findings are the first to evaluate the mechanism of SAG from the aspect of the HPA axis.

Most of the researches have concentrated on the circuits that engage in rapid signaling. However, anxiogenic responses have long been known to engage complex neuromodulatory systems, and many current therapies for anxiety disorders are based on this activity. The central systems responsible for the response to stress appear to be the NE system and the corticotropin-releasing factor (CRF) system [36]. The central monoaminergic system (including serotonin (5-HT), NE and DA) has been widely implicated in the pathophysiology and therapeutic strategies for emotional disorders [37,38]. The present study is consistent with these reports. In addition, the synthesis and release of neurotransmitters in the hippocampus and cerebral cortex are closed related to neuropsychiatric disorders [20,39]. Furthermore, the brain removed and dissected after 7 days administration is according to Lv, Li and Liu [24,40,41]. Our data shows that repeated acute restraint-stressed rats could significantly increase the concentration of NE, DA, HVA, 5-HT, and 5-HIAA in the whole cerebral cortex while NE, 5-HT, and 5-HIAA act in the hippocampus of the brain. DZP and SAG (3.6 mg/kg) could reverse this effect of repeated ARS by decreasing the concentration of NE, DA, HVA, 5-HT, and 5-HIAA in the whole cerebral cortex and NE, 5-HT, and 5-HIAA in the hippocampus of the brain.

Notably, a previous study found that the extract of *Albizzia julibrissin* (at 100 or 200 mg/kg) significantly increased time-spent and arm entries into the open arms of the EPM in rats [5], and the content of SAG in the bark of *Albizzia julibrissin* was 0.04% [6]. According to the preliminary experiments, the 3.6 mg/kg dose was chosen as the highest dose in the present study. However, our data showed that SAG exerted an anxiolytic effect only at this high dose. Testing higher doses is warranted to further elucidate the anxiolytic effect of SAG. Meanwhile, the repeated treatment of SAG for 7 days in the present study had exerted the anxiolytic effect, the effect of acute SAG will be investigated in the future. Furthermore, in order to reduce the number of animals used, and to compare the effect between the SAG and the stressed group, the SAG with the unstressed group was not designed in the present experiment, which was consistent with the previous studies [24,42]. We detected the content of neurotransmitters only in the whole cerebral cortex and hippocampus, and many researches of anxiety were also only detected the two important central regions of the brain [20,39]. Meanwhile, the amygdala plays an important role in altering monoamines such as norepinephrine and serotonin in several animal models of anxiety [43].The synthesis and release of neurotransmitters in the amygdala should also be investigated in the next step and to estimate whether the content of neurotransmitters in the amygdala is consistent with the present results. In addition, the present study proves that the mechanism of SAG involves monoaminergic systems, including NE ergic, DAergic and 5-HTergic systems, and the further progress will be made by using an antagonist.

## 4. Materials and Methods

### 4.1. Animals

A total of 60 8-week-old male Sprague-Dawley rats from Laboratory Animal Center of the Academy of Military Medical Sciences (Beijing, China) were used. Each animal was housed in individual cages in a temperature-controlled environment (22 ± 1 °C) with unlimited access to food and water. They were maintained on a 12-h light/dark cycle (light phase: 07:00–19:00). All rats were allowed to acclimatize to the standard conditions for 7 days before experimental procedures were initiated. Behavior experiments were performed between 09:00 and 14:00. The experimental procedures were approved by the Institutional Animal Care and Use Committee of the Institute of Psychology of the Chinese Academy of Sciences and in accordance with the National Institutes of Health Guide for Care and Use of Laboratory Animals.

### 4.2. Drugs and Reagents

**Figure 4:**
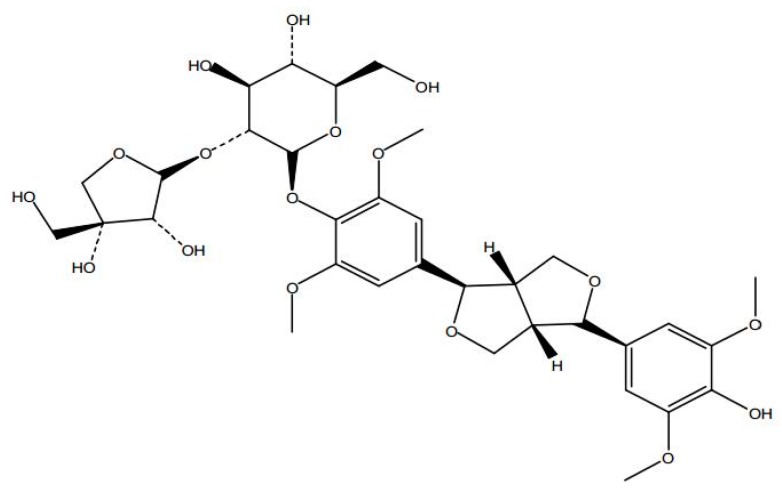
Structure of (−)-syringaresnol-4-*O*-β-d-apiofuranosyl-(1→2)-β-d-glucopyranoside (SAG).

**Figure 5:**
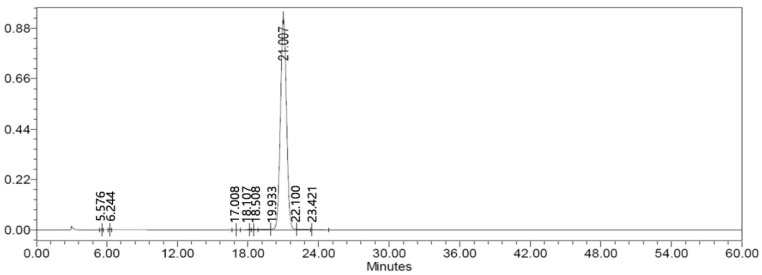
The purity of SAG detected by HPLC. SAG was analyzed by HPLC with UV detection at 204 nm. The analysis was performed with a C-18 column (4.6 mm × 250 mm) at 35 °C. It was eluted (eluent A: acetonitrile; eluent B: 0.2% phosphate solution) at a flow rate of 1.0 mL/min.

SAG (Figure 4) (PubChem CID: 91973808) was purchased from Weikeqi Biological Technology (Sichuan, China), the purity of SAG (Figure 5) was 98%, which was quantificationally analysed employing HPLC-UV method carried on a Waters 2695 HPLC system equipped with UV-vis detector.

Diazepam was obtained from Yimin Pharmaceutical Factory (Beijing, China).Norepinephrine (NE), 5-HT, 5-hydroxy-3-indoleacetic acid (5-HIAA), dopamine, 3,4-dihydroxyphenylacetic acid (DOPAC)and homovanillic acid (HVA) were purchased from Sigma (St. Louis, MO, USA). The Elisa kits of CRF, ACTH and CORT were obtained from R&D (Minneapolis, MN, USA). All of the other reagents were of analytical grade.

### 4.3. Treatment

**Figure 6:**
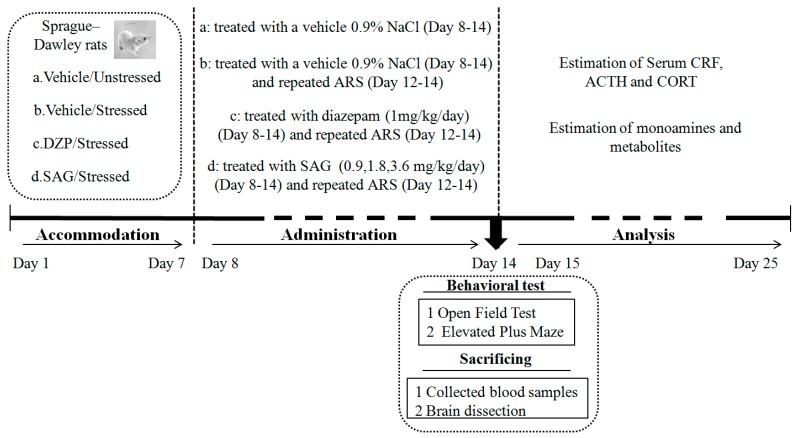
Experimental groups and procedure.

All drugs were prepared immediately before use and were given orally in a volume of 1 mL/100 g body weight. The vehicle/unstressed (V/US) group was orally administered physiological saline. Diazepam at the dose of 1 mg/kg was chosen as a positive control drug. DZP and SAG were dissolved in physiological saline. To evaluate the anxiolytic effect of SAG, the rats were orally administered SAG (0.9, 1.8, and 3.6 mg/kg) [5,6] 60 min prior to behavioral testing or diazepam 30 min before behavioral testing. The treatment protocol of doses and the administration route used for SAG and diazepam were adopted according to previous studies [6,44]. All animals were given daily administration for 7 days [5,40,41] (Figure 6).

### 4.4. AcuteRestraint Stress(ARS)

Animals were submitted to repeated restraint for 3 days by placing each rat into a plastic cylindrical restraint tube (diameter 6.5 cm, length 15 cm), ventilated by holes (1-cm diameter) that comprised approximately 20% of the tube surface. Restraint lasted for 30 min. After the last restraint, the rats were transported to the closed experiment room that was under low light, noise, and other external disturbances for 30 min.

### 4.5. Behavioral Tests

#### 4.5.1. Open Field Test (OFT)

The OFT apparatus was a 180 cm diameter cylinder with 60 cm high walls. The center of the bottom of the apparatus had a 52 cm diameter section. The rats were placed into the field at the same point against the wall and allowed to freely explore the apparatus for 5 min. The number of central entries, and the time spent in the center were recorded by an automatic video tracking system. The apparatus was thoroughly cleaned with 70% methanol after each trial.

#### 4.5.2. Elevated Plus Maze (EPM)

Immediately after the OFT, the rats were transported to the EPM room. After the adaptation of 5 min, anxiolytic activity was measured using the EPM, which consisted of two open arms (50.8 cm × 10.2 cm × 1.3 cm) and two closed arms (50.8 cm × 10.2 cm × 40.6 cm) that extended from a central platform (10.2 cm × 10.2 cm). The maze was elevated to a height of 72.4 cm above the floor. The entire maze was constructed of clear Plexiglas. Each rat was placed on the central square facing an open arm and was allowed to freely explore the maze for 5 min. Arm entries were defined as the entry of all four paws into an arm. A computer recorded the time spent on, and number of entries into the open and closed arms by means of infrared photocells. The apparatus was wiped clean with a 70% ethanol solution and dried after each subject.

### 4.6. Estimation of Serum CRF, ACTH and CORT

Immediately after the completion of the two behavioral tests, the rats were sacrificed by decapitation and blood samples were collected in EDTA coated tubes kept in ice and centrifuged at 3000 r·min^−1^ for 15 min at 4 °C. Plasma was separated and supernatants were stored at −80 °C for estimation. The contents of CRF, ACTH, and CORT were determined by a commercially available, enzyme-linked immunesorbent assay (ELISA) kit according to the manufacturer’s instructions. The absorbance of each sample was measured at a wavelength of 450 nm. The results of CRF and ACTH are presented as ng/L, and the results of CORT are presented as ng/mL.

### 4.7. Estimation of Monoamines and Metabolites

Following the collection of blood, the rats’ brains were rapidly removed and dissected, the cerebral cortex and hippocampus were isolated on an ice plate. The tissue samples were weighed and stored at −80 °C until homogenization. Levels of monoamines and metabolites (NE, DA, DOPAC, HVA, 5-HT and 5-HIAA) were estimated using High Performance Liquid Chromatography (HPLC) with an Electrochemical detector as described previously [45]. In brief, the brain tissue samples were homogenized in 0.10 Mperchloric acid by Polytron (Swedesboro, NJ, USA) homogenizer. Homogenates were then centrifuged at 1200 r·min^−1^ for 10 min at 4 °C. Twenty microliters of supernatant was injected via a HPLC pump (Model 1525, Binary Gradient Pump, Waters, Milford, MA, USA) into a column (2.1 mm ×150 mm at 30 °C, 3 μm, Waters Atlantis) connected to a Electrochemical detector (Model 2465, Waters, Milford, MA, USA) at a potential of +0.75 V with a glassy carbon working electrode Vs Ag/AgCl reference electrode. The mobile phase consists of 50 mM citric acid, 0.3 mM Na_2_-EDTA, 1.8 mM dibutylamine, and 4% methanol (pH 3.5) at a flow rate of 0.35 mL/min.

### 4.8. Statistical Analysis

The data is expressed as mean ± standard error of the mean (SEM) individual value of the rats from each group. The statistical analysis was performed using one-way analysis of variance (ANOVA), followed by the Student-Newman-Keuls post hoc test and Graph Pad Prism 5.0 software (Graphpad Sofware Inc, La Jolla, CA, USA). In cases of significant variation, the individual values were compared using Dunnett’s test. Values of *p* < 0.05 were considered statistically significant.

## 5. Conclusions

To summarize, the present data indicates that SAG induced anxiolytic-like effects on repeated acute restraint-stressed rats in the elevated plus maze and open field test. Additionally, its mechanism of action appears to be related with the HPA axis and monoaminergic systems.

## Figures and Tables

**Table 1:** Effect of SAG on the monoamines neurotransmitters and their metabolites in the whole cerebral cortex of rats.

Groups	NE (μg/g)	DA (μg/g)	DOPAC (μg/g)	HVA (μg/g)	5-HT (μg/g)	5-HIAA (μg/g)
V/US	121.35 ± 8.32	39.33 ± 5.38	35.99 ± 4.61	34.03 ± 3.58	20.31 ± 1.89	48.11 ± 3.27
V/S	188.87 ± 9.45 ^##^	65.24 ± 6.69 ^#^	49.51 ± 5.08	47.76 ± 5.15 ^#^	28.03 ± 2.43 ^#^	58.15 ± 3.92 ^#^
DZP/S	129.78 ± 4.60 **	44.88 ± 6.00 *	38.40 ± 4.26	31.32 ± 4.23 *	15.57 ± 0.97 **	31.32 ± 4.23 **
SAG/S (0.9 mg/kg)	167.45 ± 13.17	55.44 ± 6.57	45.87 ± 3.22	37.89 ± 3.41	21.14 ± 1.01 *	55.21 ± 1.84
SAG/S (1.8 mg/kg)	164.30 ± 6.12 *	52.93 ± 4.60	41.59 ± 4.01	36.47 ± 3.34	17.89 ± 1.01 **	50.92 ± 2.91
SAG/S (3.6 mg/kg)	136.10 ± 2.96 **	46.76 ± 4.44 *	40.64 ± 4.52	34.00 ± 2.97 *	17.69 ± 1.13 **	34.00 ± 2.97 **

Values are expressed as the mean ± S.E.M. # *p* < 0.05 or ## *p* < 0.01 vs. vehicle/unstressed group; * *p* < 0.05 or ** *p* < 0.01 vs. vehicle/stressed group. One-way ANOVA with Student-Newman-Keuls post hoc test.

**Table 2:** Effect of SAG on the monoamine neurotransmitters and their metabolites in the hippocampus of rats.

Groups	NE (μg/g)	DA (μg/g)	DOPAC (μg/g)	HVA (μg/g)	5-HT (μg/g)	5-HIAA (μg/g)
V/US	148.71 ± 7.94	28.36 ± 2.58	18.71 ± 1.30	19.35 ± 2.88	23.44 ± 3.24	74.29 ± 2.69
V/S	188.87 ± 9.45 ^##^	65.24 ± 6.69 ^#^	49.51 ± 5.08	47.76 ± 5.15 ^#^	28.03 ± 2.43 ^#^	58.15 ± 3.92 ^#^
DZP/S	124.13 ± 4.47 **	23.77 ± 1.20	14.78 ± 1.09	11.95±1.24	19.57 ± 1.76 **	72.54 ± 2.53 **
SAG/S (0.9 mg/kg)	153.39 ± 9.53	28.06 ± 1.96	19.93 ± 2.39	17.37 ± 1.44	25.76 ± 4.95	86.17 ± 3.88
SAG/S (1.8 mg/kg)	139.09 ± 4.89 **	28.49 ± 1.67	21.48 ± 4.66	13.28 ± 2.12	20.18 ± 1.40 *	81.13 ± 10.86
SAG/S (3.6 mg/kg)	135.37 ± 14.78 **	26.45 ± 1.24	15.93 ± 1.43	14.71 ± 1.59	19.50 ± 1.56 **	76.99 ± 4.56 *

Values are expressed as the mean ± S.E.M. # *p* < 0.05 or ## *p* < 0.01 vs. vehicle/unstressed group; * *p* < 0.05 or ** *p* < 0.01 vs. vehicle/stressed group. One-way ANOVA with Student-Newman-Keuls post hoc test.

## Supplementary Materials

Supplementary materials are available online.
